# Endocardial Endothelial Dysfunction and Unknown Polymorphic Composite Accumulation in Heart Failure

**DOI:** 10.3390/biomedicines9101465

**Published:** 2021-10-13

**Authors:** Hsuan-Fu Kuo, I-Fan Liu, Chia-Yang Li, Chien-Sung Tsai, Yung-Hsiang Chen, Wei-Shiung Lian, Tzu-Chieh Lin, Yu-Ru Liu, Tsung-Ying Lee, Chi-Yuan Huang, Chong-Chao Hsieh, Chih-Hsin Hsu, Feng-Yen Lin, Po-Len Liu

**Affiliations:** 1 Graduate Institute of Medicine, College of Medicine, Kaohsiung Medical University, Kaohsiung 807, Taiwan; medsnail@hotmail.com (H.-F.K.); chiayangli@kmu.edu.tw (C.-Y.L.); 990327kmuh@gmail.com (T.-C.L.); 2 Department of Internal Medicine, Kaohsiung Municipal Ta-Tung Hospital, Kaohsiung Medical University Hospital, Kaohsiung Medical University, Kaohsiung 807, Taiwan; 3 Department of Internal Medicine, School of Medicine, College of Medicine, Kaohsiung Medical University, Kaohsiung 807, Taiwan; 4 Division of Cardiology, Department of Internal Medicine, Kaohsiung Medical University Hospital, Kaohsiung Medical University, Kaohsiung 807, Taiwan; 5 Institute of Clinical Medicine, National Yang Ming Chiao Tung University, Taipei 112, Taiwan; Wes0208@yahoo.com.tw; 6 Heart Center, Cheng Hsin General Hospital, Taipei 112, Taiwan; 7 Division of Cardiovascular Surgery, Tri-Service General Hospital, National Defense Medical Center, Taipei 114, Taiwan; sung1500@ndmctsgh.edu.tw; 8 Department and Graduate Institute of Pharmacology, National Defense Medical Center, Taipei 114, Taiwan; 9 Graduate Institute of Integrated Medicine, College of Chinese Medicine, China Medical University, Taichung 404, Taiwan; yhchen@mail.cmu.edu.tw; 10 Department of Psychology, College of Medical and Health Science, Asia University, Taichung 413, Taiwan; 11 Core Laboratory for Phenomics and Diagnostic, Kaohsiung Chang Gung Memorial Hospital, Kaohsiung 833, Taiwan; lianws@gmail.com; 12 Department of Medical Research, Kaohsiung Chang Gung Memorial Hospital, Kaohsiung 833, Taiwan; 13 Department of Respiratory Therapy, College of Medicine, Kaohsiung Medical University, Kaohsiung 807, Taiwan; lu6525@ms42.hinet.net (Y.-R.L.); ja321cky@gmail.com (T.-Y.L.); sh12010929@gmail.com (C.-Y.H.); 14 Graduate Institute of Clinical Medicine, College of Medicine, Kaohsiung Medical University, Kaohsiung 807, Taiwan; 15 Division of Cardiovascular Surgery, Department of Surgery, Kaohsiung Medical University Hospital, Kaohsiung Medical University, Kaohsiung 807, Taiwan; 16 Department of Surgery, Faculty of Medicine, College of Medicine, Kaohsiung Medical University, Kaohsiung 807, Taiwan; 17 Department of Internal Medicine, National Cheng Kung University Hospital, College of Medicine, National Cheng Kung University, Tainan 740, Taiwan; 18 Department of Internal Medicine and Taipei Heart Institute, Taipei Medical University, Taipei 106, Taiwan; 19 Division of Cardiology and Cardiovascular Research Center, Taipei Medical University Hospital, Taipei 106, Taiwan; 20 Department of Medical Research, Kaohsiung Medical University Hospital, Kaohsiung 807, Taiwan

**Keywords:** endothelial endocardium, heart failure, mineral deposition, dilated cardiomyopathy, ischemic cardiomyopathy, unknown polymorphic composite

## Abstract

The accumulation of unknown polymorphic composites in the endocardium damages the endocardial endothelium (EE). However, the composition and role of unknown polymorphic composites in heart failure (HF) progression remain unclear. Here, we aimed to explore composite deposition during endocardium damage and HF progression. Adult male Sprague–Dawley rats were divided into two HF groups—angiotensin II-induced HF and left anterior descending artery ligation-induced HF. Heart tissues from patients who had undergone coronary artery bypass graft surgery (non-HF) and those with dilated cardiomyopathy (DCM) and ischemic cardiomyopathy (ICM) were collected. EE damage, polymorphic unknown composite accumulation, and elements in deposits were examined. HF progression reduced the expression of CD31 in the endocardium, impaired endocardial integrity, and exposed the myofibrils and mitochondria. The damaged endocardial surface showed the accumulation of unknown polymorphic composites. In the animal HF model, especially HF caused by myocardial infarction, the weight and atomic percentages of O, Na, and N in the deposited composites were significantly higher than those of the other groups. The deposited composites in the human HF heart section (DCM) had a significantly higher percentage of Na and S than the other groups, whereas the percentage of C and Na in the DCM and ICM groups was significantly higher than those of the control group. HF causes widespread EE dysfunction, and EndMT was accompanied by polymorphic composites of different shapes and elemental compositions, which further damage and deteriorate heart function.

## 1. Introduction 

According to the American College of Cardiology (ACC)/American Heart Association (AHA) guidelines for heart failure (HF), HF due to left ventricular dysfunction is categorized according to the left ventricular ejection fraction (LVEF) into HF with reduced ejection fraction (HFrEF; usually considered LVEF 40% or less) and HF with preserved ejection fraction (HFpEF) [1]. Dysfunction of the endocardial endothelium (EE) leads to the progression of HF [2]. The endocardium is the innermost layer of the heart covered with thin endothelium filled with fibrous connective tissue. Therefore, endocardial dysfunction is associated with cardiovascular diseases [3]. Typical endocardial endothelial lesions have been implicated in inflammation, thrombosis, sepsis, atrial fibrillation, ischemia/reperfusion injury, myocardial infarction (MI), cardiac hypertrophy, and HF [2,4]. The EE is a critical source of cardiomyocytes, and it can periodically release nitric oxide into the subendocardial space. Exogenous NO, or NO produced by the EE, can reduce myocardial oxygen consumption and increase myocardial perfusion, further protecting the myocardium [5,6]. In HF, high concentrations of neurohormones induce oxidative stress and cause selective damage to the EE, reducing the mechanical performance and contractility of the adjacent cardiomyocytes. Partial damage or complete loss of EE can reduce cardiomyocyte contractility and affect cardiac contractile performance [7,8]. 

The endothelial-to-mesenchymal transition (EndMT) is a process in which endothelial cells undergo several molecular transformation events and show a mesenchymal or fibroblast-like phenotype [9,10]. Thus, EndMT is an adverse reaction to disease compensation and plays a role in adaptive remodeling in a new pathological cardiac environment [10]. EndMT is associated with the development of the heart and contributes to the initiation and progression of pulmonary hypertension, atherosclerosis, valvular disease, cardiac fibrosis, and HF [11,12]. Dilated cardiomyopathy (DCM) and ischemic cardiomyopathy (ICM) are common causes of HF [13]. They have similar pathological characteristics, such as moderate to severe myofibril degeneration, vacuolation of the interstitial of the cardiac tissue, and fibrosis of cardiomyocytes through EndMT. EndMT contributes to the progression of HF through the activation of the Wnt and Snail signaling pathways and by increasing the expression of mesenchymal markers such as Wnt, β-catenin, and Snail [14,15]. In addition, accumulating evidence suggests that EndMT actively responds to valve injury, stress, and disease during heart valve development [10,16] and mediates valvular endothelial cell osteogenesis, leading to aortic valve calcification and mineral deposition [17]. 

The accumulation of composites often results in physical damage to the underlying endocardium. Currently, the known sources of endocardial deposits include lipid droplets [18], calcium [19], iron [20], fibrotic complex [21], amyloidosis [22], sarcoidosis [23], carcinoid heart disease [24], and Fabry disease [25]. Studies on composite accumulation in the damaged endocardium during HF are scarce. The mechanism underlying composite deposition during endocardium damage and HF progression is poorly understood. In this study, we aimed to analyze the elements and role of unknown composite deposition in the EE in HF progression.

## 2. Materials and Methods 

### 2.1. Heart Failure Animal Model

The animal procedures were conducted in strict compliance with Taiwanese legislation. All animal experiments were approved by the Institutional Animal Care Committee of Kaohsiung Medical University and performed according to the ARRIVE guidelines set by the National Institutes of Health for the care and use of laboratory animals (IACUC106182, 106039). Male Sprague Dawley rats (8 weeks old; weighing 240–250 g) were purchased from BioLASCO (Taiwan Co., Ltd., Taipei, Taiwan), were provided with food and water ad libitum, and maintained on hardwood bedding under a 12 h light/dark cycle. All rats were weighed weekly. All animals were housed and cared for in a pathogen-free facility at the Kaohsiung Medical University. There were two experimental groups (*n* = 6 for each group: (1) PBS (vesicle); (2) Ang II (1 mg/kg/day) (Merck, Kenilworth, NJ, USA) administered via a subcutaneously implanted Alzert osmotic pump (infusion rate, 0.5 μL/h) over 28 days [26]; and (3) left anterior descending coronary artery (LAD) ligation for 28 days [27]. After 28 days, the rats were euthanized with CO_2_ and their hearts were immediately harvested for further analysis.

### 2.2. Scanning Electron Microscopy 

The collected cardiac tissues were fixed overnight in 2.5% glutaraldehyde at 4 °C. The tissues were washed three times with 1× phosphate-buffered saline (PBS) for 10 min, post-fixed in 2% osmium tetraoxide (OsO_4_) for 2 h at 4 °C, washed three times with PBS for 10 min, and dehydrated using ascending grades of alcohol (50%, 75%, 85%, 95%, and 100%). The tissues were dried using a critical point drier (CPD 030; Bal-TEC, Pfäffikon, Switzerland) for 1 h, and the tissues were coated with gold and monitored by SEM (Hitachi-8010; Hitachi, Tokyo, Japan) at an accelerating voltage of 10–25 kV.

### 2.3. Energy-Dispersive X-ray Spectroscopy (EDS) 

The existence and percentage of ions in the cardiac tissue samples were examined using an SEM equipped with an energy dispersive X-ray spectrometer. The SEM system (cold field emission SEM) (JSM-7000F, JEOL; Akishima, Tokyo, Japan) contained a scientific-grade high-resolution in-lens detector equipped with variable pressure and energy dispersive X-ray spectrometer for characterizing the elemental composition of a specimen. Imaging and elemental analyses were carried out in a specimen chamber filled with nitrogen gas at a variable pressure of 60 Pa with an accelerating voltage of 17.5 kV using INCA (Oxford Instruments, Abingdon, UK) software for the EDS analysis (peak identification, elemental composition assessment, and processing of the measured signal). For intracellular ion assessment, Point & ID (a feature of the INCA software) (Oxford, High Wycombe, UK) was used to specify a rectangular region of interest covering the sediments, identify spectral peaks, and determine the percentage of the identified elements.

### 2.4. Human Sample Collection 

We collected samples from 11 subjects with HF, six with DCM, five with ICM, and three healthy donor auricle tissues (from coronary artery bypass grafting patients, Table S1). All end-stage HF patients were treated with heart transplant surgery in the Tri-Service General Hospital in Taipei from August 2018 to July 2020. Furthermore, the research protocol for the clinicopathological assessment of end-stage HF in explanted heart inpatients was reviewed and approved by the institutional review board (IRB) of the Tri-Service General Hospital (TSGHIRB No: 2-106-05-141).

### 2.5. Immunohistochemistry Staining

To quantify CD31, E-cadherin, VE-cadherin, Tropomyosin, and β-catenin expression, tissue sections were incubated in blocking buffer (0.5% bovine serum albumin, BSA, 0.05% Tween-20, and PBS) for 1 h, followed by blocking with specific primary antibodies: CD31 (1:100, sc53411; Santa Cruz, Santa Cruz, CA, USA), E-cadherin (1:100, sc8423; Santa Cruz), VE-cadherin (1:100, sc9989; Santa Cruz), β-catenin (1:100, GTX101435; Gene Tex, Irvine, CA, USA), and tropomyosin (1:100, sc58868, Santa Cruz) for 1 h. Antibody staining was performed using a fluorescence detection system (Ventana Medical System; Invitrogen, Carlsbad, CA, USA) for 1 h. After washing, the sections were mounted and examined using a confocal laser microscope (Leica, Baca Raton, FL, USA). 

### 2.6. Statistical Analysis

The data are presented as mean ± standard error of mean (SEM) and analyzed using the ANOVA and Dunnett’s tests. Statistical analyses were performed using SigmaStat version 3.5 (Systat Software Inc., Chicago, IL, USA), and the results with *p* < 0.05 were considered statistically significant.

## 3. Results 

### 3.1. HF-Induced EE Injury Led to Myocardium Myofibril Fragmentation and Mitochondrial Rearrangement

**Figure 1:**
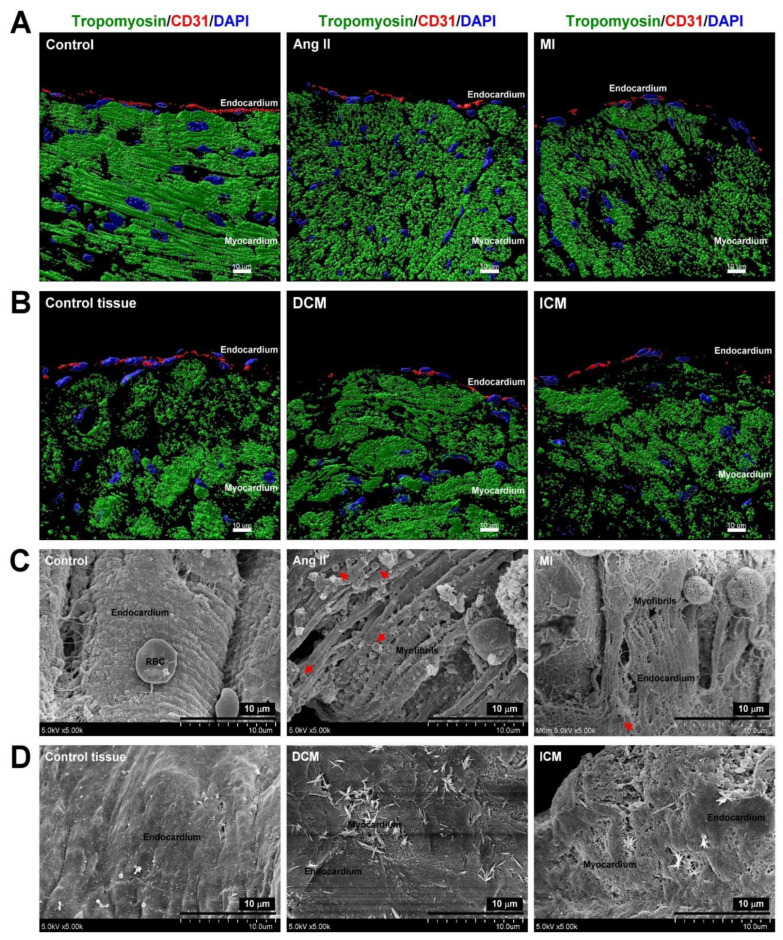
Endocardial endothelial dysfunction in HF. Immunostaining of CD31 (endocardium marker, red), tropomyosin (myocardium marker, green), and DAPI (DNA, blue) in rat HF heart model (**A**) and human HF heart tissues (**B**). Scanning electron microscopy (SEM) images of rat HF heart model (**C**) and human HF heart section (**D**). Red arrow indicates the mitochondria. Scale bar: 10 μm.

Irrespective of whether it is hypertensive or ischemic HF, EE dysfunction initiates the progression of HF [28]. We established animal models of HF induced by Ang II (hypertensive HF) and MI (ischemic HF) treatment. We also collected non-HF atrial specimens and specimens of HF caused by DCM and ICM for further examination. SEM data demonstrated that the endocardium in the control group was intact and that there was no damage or cracking. In contrast, the endocardium in the MI and Ang II groups showed severe myofibril damage and fracture with a lower number of mitochondria filled between the broken myofibrils. In addition, the HF group also had an unidentified composite deposited on the damaged endocardium (Figure 1A). In the human specimen, we found that the DCM and ICM groups had apparent endocardial damage and were accompanied by the deposition of unknown composites on the EE (Figure 1B). Next, immunostaining was used to confirm whether the integrity of the endocardial structure was damaged during HF. The expression of CD31 (EE marker protein) was significantly reduced in the animal and human HF groups (Figure 1C,D) indicating EE dysfunction, which causes myofibril fragmentation, mitochondrial dysfunction, and unidentified composite deposition.

### 3.2. HF-Induced Endothelial-to-Mesenchymal Transition in the Endocardium

**Figure 2:**
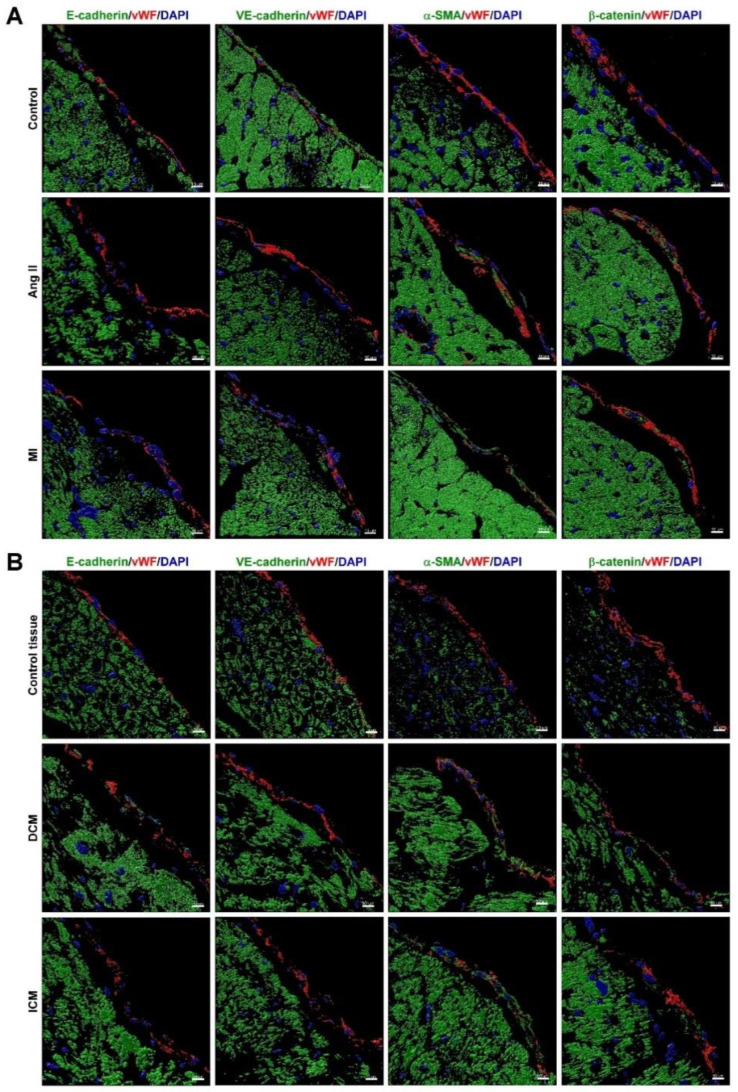
Endothelial-to-mesenchymal transition (EndMT) in the endocardium in HF. Immunostaining of vWF (endocardium marker, red), E-cadherin (green), VE-cadherin (green), α-SMA (green), β-catenin (green), and DAPI (DNA, blue) in the rat HF heart model (**A**) and human HF heart section (**B**). Scale bar: 10 μm.

EndMT is a pathological process in which fibroblast-like cells replace the original endothelial cells. It is common after endocardial ischemia or damage due to the disease. This process is also essential for the progression of HF [11]. Figure 2 shows the HF-induced EndMT in the endocardium. Immunofluorescence labeling of the control group showed a higher expression of endothelial-specific marker von Willebrand Factor (vWF) combined with the endothelial marker E-cadherin and VE-cadherin in the endocardium; however, the mesenchymal markers α-SMA and β-catenin were not upregulated. In the HF groups, mesenchymal markers α-SMA and β-catenin were highly expressed in combination with vWF, significantly reducing the expression of endothelial markers E-cadherin and VE-cadherin. These data show that HF induced EndMT in human and mouse endocardial endothelial cells.

### 3.3. Unknown Composite Accumulation in the Endocardium of the HF Model

**Figure 3:**
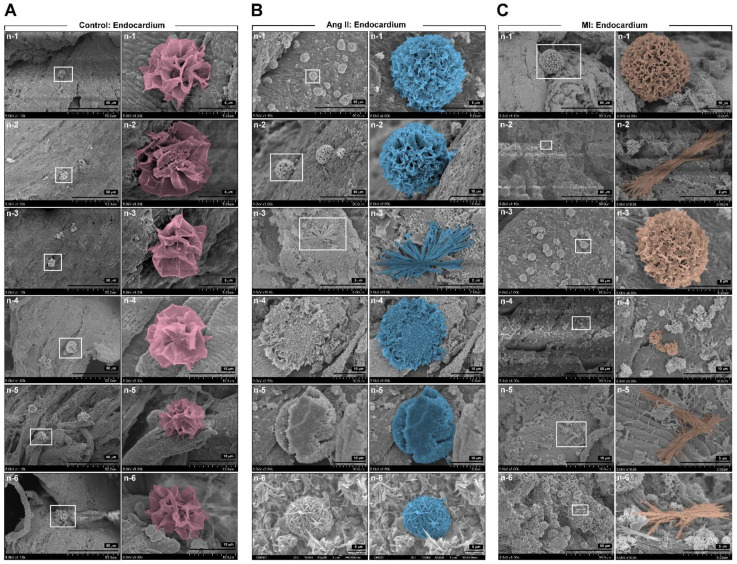
Scanning electron microscopy images of mineral deposition in Ang II- and ischemia heart-induced HF model. (**A**) Control group endocardium. (**B**) Ang II group endocardium. (**C**) LAD ligation-induced MI group endocardium.

Research on the deposition of unknown substances in the endocardium in HF diseases is limited. Figure 3 shows the deposition of unknown substances in the endocardium. The HF samples showed the deposition of multiform unknown composites, including flower-like structures (in the NR group) (Figure 2A). In the HF group, the appearance of multiform unknown composites included sponge-like, hedgehog-like, mushroom-like, grass-like, brushwood-like, ball containing stingers such as asparagus-like, underbrush-like, scarecrow-like, moss-like, star-like, and hydrangea-like structures (Figure 2B,C). These data showed that the endocardial surface covering may have unknown composite accumulation of different shapes, especially in HF compared with the control, and that the composition elements and functions may vary.

### 3.4. Elemental Analysis of Unidentified Composite on the Endocardium in HF Models 

**Figure 4:**
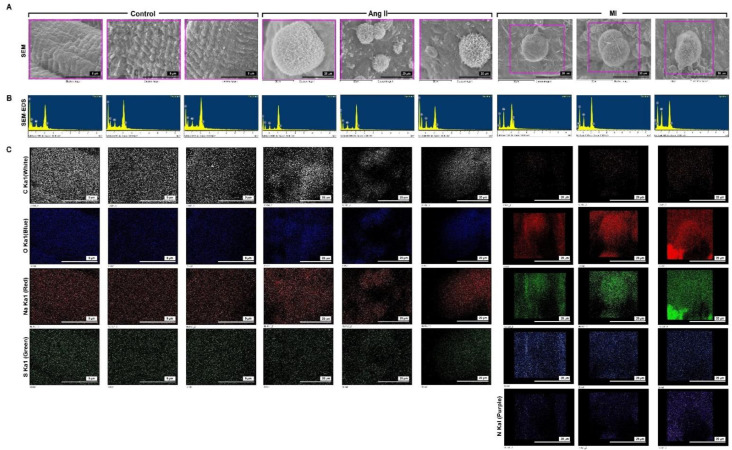
SEM/energy dispersive x-ray spectrometry (SEM/EDS) elemental mapping of the mineral deposition in the Ang II- and ischemia heart-induced HF models. (**A**) SEM images of HF animal model. (**B**) SEM-EDS mapping. (**C**) Secondary electron image and analogous elemental mapping of carbon (C), oxygen (O), sodium (Na), sulfur (S), and nitrogen (N).

SEM, ESEM-EDS, and EDS mapping were used to characterize the unknown composite elements in the HF endocardium (Figure 4). The surface topography of a partial cross-section of unknown composite deposit was assessed by SEM (Figure 3A). ESEM-EDS and EDS mapping images of C, O, Na, S, and N are shown in Figure 3B–3D. The corresponding C, O, Na, S, and N bright spots indicate mineral-like sediments and illustrate the distribution of these elements. The unknown composite element weight percentage and atomic percentage were analyzed in the three groups (Table 1). The unknown composite element percentage in the control group was C > O > Na > S. Unknown composite element percentage in the Ang II group was C > O > Na > S. Unknown composite element percentage in the MI group was O > C > Na > N > S. Our findings indicate that the weight percentage and atomic percentage of C were significantly reduced in the MI group and there was little decrease in the Ang II group. The percentage of O and Na increased in the HF group, especially in the MI group. The weight percentage and atomic percentage of S were only reduced by Ang II, whereas N was detected only in the composite of the MI group. These data showed that the composition and ratio of the sedimentary materials of the control group and different HF groups were variable. Hypertensive HF increases the O and Na elements. However, in the ischemic HF group, the percentage of O, Na, and N was significantly increased, and the ratio of C decreased in the current residence. This result indicates a correlation between the increase in the S/N concentration caused by ischemic HF and the production of composites.

### 3.5. Unidentified Composite Accumulation on Endocardium in Human HF 

**Figure 5:**
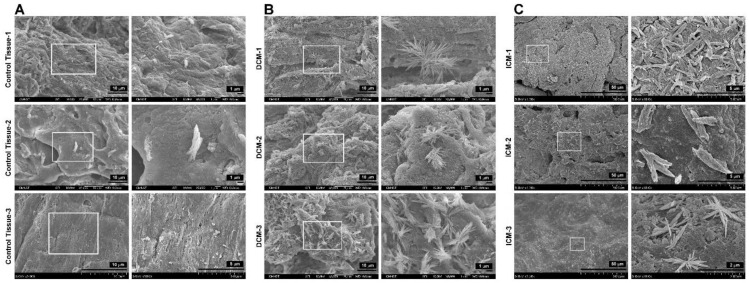
Mineral deposition in healthy subjects and patients with DCM and ICM. (**A**) Depositions in the right auricle endocardium from coronary artery bypass graft (CABG) surgery patients (non-HF). (**B**) Depositions in the endocardium of patients with DCM. (**C**) Depositions in the endocardium of patients with ICM.

Figure 5 shows the accumulation of unknown substances in the human HF endocardium observed by SEM. A small number of deposits that looked like stalactites or stalagmites were found in the endocardium of the right atrial appendage without HF (in control tissue groups 1–3) (Figure 5A). In the DCM HF group, a significant accumulation in the endocardium was found, and it resembled bushes or crystal clusters (Figure 5B). In the ICM HF group, many accumulations of lichen or crystal clusters were found in the endocardium (Figure 5C). The results of this study and the pattern of deposits in the animal HF model confirm that the endocardium in humans without HF may have a small number of stalactite-like deposits, whereas the composites on the endocardium in the HF group might appear as bushes or lichens. 

### 3.6. Elemental Analysis of Unidentified Composite on Endocardium in Human HF 

**Figure 6:**
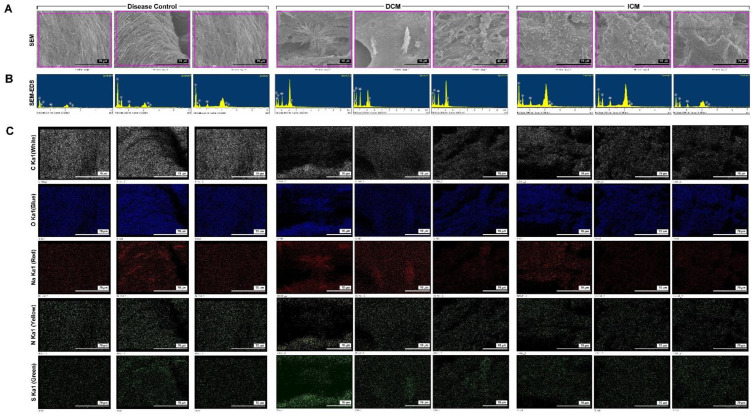
SEM/ED mapping of the mineral deposition in healthy subjects and patients with DCM and ICM. (**A**) SEM image of mineral deposition in HF patients. (**B**) SEM-EDS mapping. (**C**) SEM-EDS elemental analysis.

Figure 6 shows the SEM, ESEM-EDS, and EDS mapping data used to characterize the elements in the unknown composite in the human HF endocardium. Figure 6A shows the surface topography of a partial cross-section of the unknown composite deposit assessed by SEM. ESEM-EDS and EDS mapping images of C, O, Na, N, and S in unknown composites are shown in Figure 6A–C. The element weight percentage and atomic percentage of the unknown composites were analyzed in the three groups (Table 2). The proportion of elements in the unknown composites in the control tissue group was O > C > N > Na > S; DCM group was O > C > Na > N > S; and ICM group was O > C > N > Na > S. Our findings indicated that the weight percentage and atomic percentage of C were significantly increased in the HF group, and there was no difference between the DCM and ICM groups. The weight percentage and atomic percentage of N were only reduced in the DCM group. The weight percentage and atomic percentage of O and Na were increased in the HF group; however, there was no difference between the DCM and ICM groups. The Na concentration increased significantly in the DCM group, which was different from that in the other two groups. The weight percentage and atomic percentage of S significantly increased in the HF group. This finding indicates a correlation between the increase in Na/S concentrations caused by HF and the production of composites. 

## 4. Discussion

Cardiomyopathies are an important and heterogeneous group of diseases that contribute to HF [29]. The main types of primary cardiomyopathy are hypertrophic cardiomyopathy (HCM), DCM, restrictive cardiomyopathy, arrhythmogenic right ventricular dysplasia, and transthyretin amyloid cardiomyopathy [30]. HCM is the most common primary cardiomyopathy (prevalence of 1:500) [31], and the presence of DCM (prevalence of 1:2500) primarily indicates the need for heart transplantation [32]. Ischemic cardiomyopathy (ICM) is the most common type of DCM and is an essential etiology of HF associated with a heart attack or coronary artery disease (CAD). In this study, the human HF samples were collected from DCM and ICM heart transplant recipients.

Animal models that mimic human HF, such as myocardial infarction (LAD ligation)-induced HF and pressure-overload (Ang II treatment)-induced HF, are standard models to study cardiac fibrosis and HF progression [33]. Interstitial fibrosis is an essential pathological process in human HF. Compared with the extensive interstitial fibrosis in the Ang II model, the cardiac fibrosis caused in the TAC model is less rapid, and the effect is not systemic or extensive [34,35]; therefore, to establish a model of non-ischemic HF, we used Ang II instead of the transverse aortic constriction (TAC). 

EE dysfunction plays an essential role in facilitating HF and has a significant prognostic value for clinical outcomes. Previous studies have indicated that biomarkers of EE, such as platelet endothelial cell adhesion molecule (PECAM-1/CD31), vascular cell adhesion molecule-1 (VCAM-1), soluble thrombomodulin, and von Willebrand factor (vWF) [2], were significantly decreased in HF. Our experimental results indicated that hypertensive or ischemic HF directly damages the integrity of the EE with reduced CD31 expression, leading to the fragmentation of the myocardial fibers under the endocardium and exposure of the mitochondria initially located in the myocardium. 

EndMT is a complex pathological progression in which the original features of endothelial cells (EC) are lost, and they develop mesenchymal features of fibroblasts or myofibroblasts during embryonic or cardiac disease remodeling [36]. Recent studies have demonstrated that EndMT may be an important pathogenic mechanism in fibrotic disorders, including pulmonary, kidney, cardiac fibrosis, and HF [37]. The process of heart remodeling during HF often involves the activation of several ECs and mesenchymal cell transition-related pathways. These pathways include the cell–cell junction reconstruction [38], increased nuclear factor-κB (NF-κB) transcription factor activity [39], activation of the transforming growth factor β (TGF-β)/fibroblast growth factor (FGF) axis coordinating the endothelial cell plasticity and smooth muscle cell migration motility [40], activation of the Smad2/3-Snail signaling pathway to increase EndMT protein expression [41], and the regulation of microRNA (miRNA) expression for a positive or negative mediation of HF progression [42]. Our results showed that hypertensive or ischemic HF significantly reduced the E-cadherin and VE-cadherin expression and increased vimentin and fibronectin expression in the human and rat HF endocardium. In addition, our research showed that during hypertensive and ischemic HF, EE is damaged, accompanied by EndMT, indicating that the EE damage and EndMT play an essential role in the progression of HF.

EndMT contributes to extracellular matrix remodeling and collagen-deposition-induced cardiac fibrosis [43]. However, we found that the endocardial deposits in HF are significantly different from the currently known endocardial or valve deposits. The vascular calcification, valve calcium, mineral deposits or the formation of calcium phosphate complexes in the endocardium layer on the vasculature or valve are the most common endocardial deposits [44]. Vascular calcification or valve calcium is associated with chronic kidney disease, hypertension, hyperphosphatemia, aging, and diabetes mellitus [45]. Unlike the contents of the cardiac deposits formed in other diseases, the deposits found in the HF groups in our experiments were organic complexes rich in Na. To the best of our knowledge, this is the first report related to the study of endocardial deposits. We found various types of composites on the surface of the HF endocardium but not on the normal endocardium. In the normal group, the endocardium was complete and smooth, and there were few cases of endocardial damage. However, the number of composites was small, and mostly flower-like. In the HF group, the endocardium was damaged, myocardial fibers were fragmented, and various types of deposits were intertwined and stacked on the endocardium. However, the appearance of the composites in the HF animal models was significantly different from that of the composites in the human HF heart sections. Regardless of Ang II or ischemic HF, the composites were generally enormous and had a solid sponge-like structure. There were not many differences between the two HF animal models. The composites in the human HF groups were mostly shrub, stalk, or crystal shaped.

Elemental analysis shown many deposits of different shapes rich in Na on the endocardium of HF. In the animal model of HF, compared with the other two groups, the MI group presented significantly reduced C concentration, whereas the percentage of O and Na was significantly increased. It is worth mentioning that the MI group was the only group with deposits containing N, whereas the concentration of S did not differ among the three groups. Our findings showed a correlation between the increase in Na and N concentrations in the ischemic HF group. In the Ang II group, the C and S concentrations in the composites marginally decreased compared with those in the control group except for an increase in the Na and O concentrations. In human HF heart sections, the composites of the ICM group showed a reduction in O concentration and an increase in C, S and Na concentrations. This trend was not like that observed in the MI group in the animal experiments. However, the concentration of N was not different in the composites among the three tissues. In the DCM group composites, the percentage of O decreased, whereas that of C, Na, and S increased. Our finding showed a correlation between the increase in Na and S concentrations in the DCM group.

The deposits in the DCM and ICM groups contained a significantly higher concentration of Na than those in the other groups. However, the reasons for the formation of Na-rich composites in the damaged endocardium, the pathological mechanism, and the effect on the clinical prognosis of HF have not been explored to date. The non-osmotic arginine vasopressin (AVP), the renin-angiotensin-aldosterone axis, and the sympathetic nervous system activate vascular resistance and enhance sodium and water renal retention, leading to hyponatremia in hospitalized patients with HF [46]. Hypervolemic hyponatremia (serum sodium concentration < 135 mEq/L) is a frequent electrolyte imbalance encountered with poor short- and long-term clinical outcomes (1.5–1.7-fold increased risk of 30-day mortality) in hospitalized patients with HF [47,48]. However, it is unclear whether there is a direct correlation between hyponatremia and the accumulation of Na-rich composite in the endocardium in HF; therefore, further research is warranted.

Our study had a few limitations. First, owing to the difficulty of obtaining patient specimens, the tissue specimens used in the normal group were from the right atrial appendage and not ventricular tissue. However, this does not affect the integrity of the endocardium, EndMT, or deposition. In the evaluation of physical performance, compared with other HF groups, morphological differences still exist in the structure of the ventricles. Furthermore, EE dysfunction and EndMT activation are required for HF progression. However, culturing the ventricular endocardial endothelial cell line is challenging, limiting the research needed to understand the pathological mechanism. This may be solved using the rat primary ventricular endocardial endothelial cells. Our results showed that the accumulation of sodium-rich composites, EE dysfunction, and EndMT activation were indispensable in the progression of HF. However, the use of these three phenomena for predicting HF progression is still not established. The sodium-rich composite deposition can be detected using 3D imaging, SEM, and EDS analysis. However, this experimental strategy cannot be used in the early stages of HF diagnosis or disease progression. Developing new imaging detection systems may overcome these limitations. HF is often accompanied by hyponatremia, and the composites on the endocardial surface accumulated primarily in the damaged part of the endocardium. Importantly, in this study, the level of Na in the plasma was not measured regardless of animal or human model results. Overall, we demonstrated that hypertensive and ischemic HF leads to EE dysfunction and EndMT and that the deposition of unknown composites in the endocardium promotes HF progression.

## 5. Conclusions

**Figure 7:**
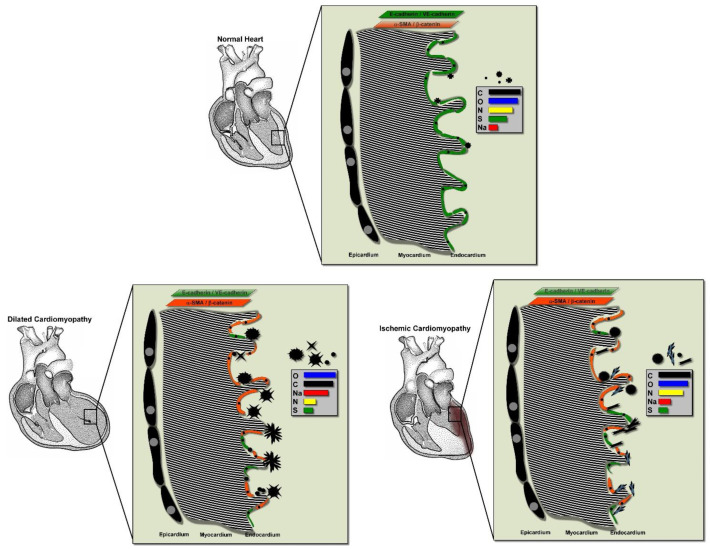
Schematic representation of the mechanism of HF-induced composite accumulation in the endocardium. In a normal heart, the endocardium is integrated and smooth, and the number of composites is small. In the HF group, the endocardium is damaged and EndMT is induced. The fragmented myocardial fibers and various types of deposits are intertwined and stacked on top of the endocardium.

In summary, our study demonstrated that HF induces composite accumulation in the endocardium in association with EE dysfunction and EndMT. Polymorphic composites differ in shape and elemental composition and can further damage and deteriorate heart function. In the animal HF model, the weight and atomic percentages of Na were significantly higher than those of the control group, especially HF caused by MI-induced Na/N-rich composites. The deposited composites in the human HF tissue showed a significantly higher percentage of Na and S than those in the control group, especially DCM HF-induced Na/S-rich composites (Figure 7). 

## Figures and Tables

**Table 1:** Quantification of area spectrum for the weight and atomic percentage of various elements in Ang II- and ischemia heart-induced HF models. * *p* < 0.05; ** *p* < 0.001.

Element		Control	Ang II	MI
C	Weight%	61.83 ± 4.81	53.8 ± 1.61 *	4.47 ± 0.59 **
	Atomic%	71.31 ± 4.38	63.27 ± 1.26 *	6.48 ± 0.85 **
O	Weight%	25.27 ± 4.24	33.236 ± 0.31 *	59.31 ± 1.39 **
	Atomic%	21.93 ± 3.98	29.35 ± 0.34 *	64.52 ± 1.46 **
Na	Weight%	6.88 ± 0.56	9.51± 1.30 *	25.72 ± 3.2 **
	Atomic%	4.554 ± 0.4	6.713 ± 0.85 *	21.884 ± 2.41 **
S	Weight%	6 ± 0.9	3.44 ± 0.47 *	5.03 ± 2.06
	Atomic%	2.59 ± 0.42	1.52 ± 0.21 *	2.73 ± 1.13
N	Weight%	ND	ND	5.46 ± 1.1 **
	Atomic%	ND	ND	6.79 ± 0.77 **

ND: not detectable.

**Table 2:** Quantification of area spectrum for the weight and atomic percentage of various elements in DCM and ICM. * *p* < 0.05; ** *p* < 0.001.

Element		Control Tissue	DCM	ICM
O	Weight%	20.22 ± 4.75	30.08 ± 3.42 *	30.79 ± 3.99 *
	Atomic%	17.25 ± 4.29	29.13 ± 3.69 *	27.66 ± 3.91 *
C	Weight%	54.96 ± 5.8	34.29 ± 2.53 **	42.77 ± 1.73 *
	Atomic%	62.32 ± 5.86	44.15 ± 2.59 **	51.23 ± 1.28 *
Na	Weight%	4.3 ± 0.67	13.17± 1.04 **	7.29 ± 0.93 **
	Atomic%	2.94 ± 0.39	9.69 ± 0.81 **	5.19 ± 0.63 **
S	Weight%	3.82 ± 0.73	11.15 ± 1.63 **	5.03 ± 2.24 *
	Atomic%	1.62 ± 0.31	5.38 ± 0.35 **	2.26 ± 0.13 *
N	Weight%	16.69 ± 4.63	11.29 ± 3.97	14.06 ± 6.17
	Atomic%	16 ± 2.51	12.45 ± 2.22	14.38 ± 3.27

## Data Availability

Data will be provided on request.

## Supplementary Materials

The following is available online at https://www.mdpi.com/article/10.3390/biomedicines9101465/s1, Table S1: Clinical characteristics of CABG patients.
