# Supplementary material for: Endocardial Endothelial Dysfunction and Unknown Polymorphic Composite Accumulation in Heart Failure
Source: Biomedicines. 2021 Oct 13;9(10):1465. doi: 10.3390/biomedicines9101465 (PMC8533412; doi:10.3390/biomedicines9101465)
Supplement: Supplementary file 1 [file biomedicines-09-01465-s001.zip › biomedicines-1360609-supplementary.pdf]

Endocardial endothelial dysfunction and unknown polymorphic composite accumulation in heart failure

Supplementary File

Table S1. Clinical characteristics of CABG patients.

Characteristics	Patient 1	Patient 2	Patient 3
Male	Y	Y	N
Age (years)	56	54	57
Diabetes	Y	Y	N
Hypertension	Y	Y	Y
Hyperlipidemia	Y	Y	Y
Smoking	Y	Y	N
Alcohol	N	Y	N
Diagnosis	3VD	3VD	3VD+LMCA
NYHA Functional Class	II	II	II
CCS Score	1	1	1
Euro Score II Score	0.0079	0.0075	0.0075
LVIDd (cm)	4.95 cm	4.68 cm	5.51 cm
LVEF (%)	0.659	0.78	0.807
CO (L/min)	6.697 L/min	4.819 L/min	7.884 L/min
LV Mass (gm)	198 gm	137 gm	267 gm

Abbreviations: 3VD, Three-vessel disease; LMCA, left main coronary artery; CCS score, Coronary calcium score; LVIDd, left ventricular dimensions at end-diastole; LVEF, left ventricular ejection fraction; CO, cardiac output.
